# Prevention of salivary gland dysfunction in patients treated with radioiodine for differentiated thyroid cancer: A systematic review of randomized controlled trials

**DOI:** 10.3389/fendo.2022.960265

**Published:** 2022-08-29

**Authors:** Arunrat Auttara-atthakorn, Jaruwan Sungmala, Thunyarat Anothaisintawee, Sirimon Reutrakul, Chutintorn Sriphrapradang

**Affiliations:** ^1^ Division of Endocrinology and Metabolism, Faculty of Medicine Ramathibodi Hospital, Mahidol University, Bangkok, Thailand; ^2^ Division of Endocrinology and Metabolism, Faculty of Medicine, HRH Princess Maha Chakri Sirindhorn Medical Center, Srinakharinwirot University, Nakornnayok, Thailand; ^3^ Department of Family Medicine, Faculty of Medicine Ramathibodi Hospital, Mahidol University, Bangkok, Thailand; ^4^ Division of Endocrinology, Diabetes and Metabolism, Department of Medicine, University of Illinois at Chicago, Chicago, IL, United States

**Keywords:** parotid gland, prophylaxis, radiation effects, radioactive iodine, sialadenitis, thyroid cancer, xerostomia

## Abstract

**Introduction:**

Salivary gland dysfunction (e.g., sialadenitis and xerostomia) is the most common complication of radioactive iodine (RAI) therapy for differentiated thyroid cancer (DTC). Several methods have been used to reduce/prevent this adverse effect. We aimed to systematically review the effectiveness of non-pharmacological and pharmacological interventions in preventing RAI-induced salivary gland dysfunction in patients with DTC.

**Methods:**

A systematic review was conducted, according to PRISMA guidelines. The protocol was registered (PROSPERO: CRD42022295229). PubMed, Embase, Scopus, and the Cochrane Library electronic databases were searched from inception to November 2021. Inclusion criteria were randomized controlled trials of DTC patients who were older than 18 years and underwent RAI after thyroidectomy in which at least one studied group received an intervention to prevent salivary gland dysfunction.

**Results:**

Twelve studies (a total of 667 participants) were included. Among DTC patients who were treated with RAI, nonpharmacological treatment such as parotid gland massage and aromatherapy ameliorated salivary gland dysfunction. Antioxidants such as vitamin E and selenium demonstrated radioprotective effects on the salivary gland, while other antioxidants did not show radioprotective benefits. Vitamin C showed no significant effects on preventing salivary gland dysfunction. Amifostine had inconsistent outcomes among studies. Among cholinergic agonists, pilocarpine did not demonstrate the radioprotective effect on parotid glands, while bethanechol lowered salivary gland dysfunction. However, the negative results from pilocarpine may be explained by the strong sialorrheic effect of the Cincinnati regimen in both study arms.

**Conclusion:**

Among non-pharmacological and pharmacological methods, parotid gland massage, aromatherapy, vitamin E, selenium, amifostine, and bethanechol may have benefits in minimizing RAI-induced salivary gland dysfunction in patients with DTC. The results are limited by a small number of patients and should be confirmed in future larger randomized controlled trials.

**Systematic Review Registration:**

https://www.crd.york.ac.uk/prospero/display_record.php?RecordID=295229, PROSPERO, identifier CRD42022295229.

## Introduction

Radioactive iodine (RAI) therapy with iodine-131 (I-131) is administered after thyroidectomy in patients with differentiated thyroid cancer (DTC) to ablate normal thyroid tissue and treat residual tumors and metastases through its beta-emitting action (1). Its use is based on the unique ability of the thyroid follicular cells to concentrate iodide through the membrane sodium-iodide symporter. The adjuvant RAI provides an effective treatment of DTC which contributes to minimized risk of disease recurrence and metastasis (2).

One of the most common complications of RAI therapy is salivary gland dysfunction (sialadenitis, xerostomia, decreased or altered sense of taste, and dental caries) because the salivary glands can also physiologically uptake iodide through the sodium-iodide symporter. The high concentration of I-131 in the salivary gland could be about 30 to 40 times compared to plasma levels (3). The effects of I-131 can cause direct injury to the intralobular ductal epithelium of salivary glands resulting in periacinoductal inflammation and fibrosis. Consequences of inflammation from I-131 include endothelial injury and increased vascular permeability. Acute sialadenitis is characterized by pain and swelling of the salivary gland caused by duct obstruction, mucous retention, and increased periductal pressure. The narrowing of the duct lumen is secondary to the inflammatory process (4). It usually presents as bilateral involvement, most commonly in the parotid gland (4, 5). The sialadenitis of the parotid gland is more common than that of the submandibular gland. This is because the parotid gland has a higher proportion of serous cells that are more sensitive to I-131 than the submandibular gland that has a higher proportion of mucous acini (4, 6, 7). However, a more recent paper by Barrueco et al. found that RAI-induced sialadenitis frequently involved unilaterally and occurred equally in both parotid and submandibular glands (8). The sialadenitis begins within 24-48 hours and persists for a few days (9). These effects can develop months or years later and can persist leading to chronic glandular changes (4). Incidence of acute sialadenitis varies with a range of 2-67% (10) and that of chronic sialadenitis ranges from 11 to 43% (4). The higher cumulative dose of radioiodine in the salivary gland is associated with an increased risk of salivary gland damage (6–8, 11). These adverse effects have a great impact on patients’ quality of life. Salivary gland damage/dysfunction can be quantified by technetium-99m pertechnetate salivary gland scintigraphy (SGS) (12), sialography, saliva flow rate (13), measurement of serum amylase (14), and serum oxidative stress markers (15). The major advantage of SGS is that it assesses both uptake function and excretion fraction of all four major salivary glands (12).

Potential prophylactic interventions include hydration, use of sialogogues (such as lemon juice, sour candy, and chewing gum), parotid gland massage, amifostine, and cholinergic agents (1, 2). However, there is no consensus on recommended methods for the prevention of salivary gland dysfunction. A previous systematic review of the non-pharmacological management to diminish salivary gland dysfunction after RAI therapy for DTC showed that parotid gland massage and sialogogues demonstrated the greatest reduction in salivary gland dysfunction, but the included studies were limited in number, sample size, the strength of evidence, and generalizability (16). To better inform clinicians regarding the effectiveness of salivary gland protective methods, we aimed to systematically review randomized controlled trials (RCTs) that assessed the effects of both non-pharmacological and pharmacological methods in preventing RAI-induced salivary gland dysfunction in patients with DTC. We also discussed the established salivary gland protective methods from the radiation treatment of head and neck cancers compared with RAI treatment in patients with DTC.

## Methods

The study was conducted according to the Preferred Reporting Items for Systematic Reviews and Meta-Analyses (PRISMA) guidelines. The review protocol was prospectively registered with PROSPERO International prospective register of systematic reviews (CRD42022295229).

### Data sources and search strategy

An initial search was conducted in the following electronic databases: PubMed (MEDLINE), Embase, Scopus, and the Cochrane Library, from inception to November 2021. Additionally, the citations of relevant studies were also searched. The keywords used were combinations of “radioiodine”, “radioactive iodine”, “iodine-induced”, “¹³¹I”, “sialadenitis”, “sialoadenitis”, “salivary gland”, “prevention”, “protection”, “aromatherapy”, “lemon candy”, “vitamin E”, “vitamin C”, “massage”, “selenium”, “amifostine”, “pilocarpine”, and “chewing gum.” The systematic search strategies are available in the **Supplementary Data (Appendix 1)**.

### Eligibility criteria and study selection

Studies were considered eligible according to the following criteria: (1) RCTs; (2) participants with DTC who were > 18 years old; (3) RAI therapy was given following total or near-total thyroidectomy; (4) at least one studied group received an intervention with the purpose to reduce salivary gland dysfunction; (5) reported outcomes regarding salivary gland dysfunction such as xerostomia, sialadenitis, SGS, and (6) reports were published in English. Other publication types, such as reviews, meta-analysis, case reports, and conference abstracts were excluded. Two reviewers (AA and JS) independently selected eligible studies by screening the title and abstract and assessing the full texts. Any discrepancy was resolved by consensus or by another reviewer (CS).

### Study outcomes

The primary outcomes were adverse events to the salivary gland resulting from RAI therapy. These adverse events included xerostomia, sialadenitis, change in quality of life, change of serum amylase, change of serum oxidative stress markers, and reduction in salivary gland function which was quantitatively evaluated by SGS.

### Data extraction and quality assessment

Extracted data included information on the first author, year of publication, country, eligible criteria, RAI dose, allocation of interventions, additional intervention, and outcome measurements. Two reviewers (AA and JS) independently collected the data from each study, followed by a cross-check of consistency. The quality of the included study was independently assessed using the RoB2, a revised tool for assessing the risk of bias in randomized trials (17). The quality aspects assessed by this scale included randomization, deviations from the intended interventions, missing outcome data, measurement of the outcomes, and selection of reported results (**Supplementary Data, Appendix 2**). Discrepancies were resolved by re-evaluation and discussion with another reviewer (CS).

## Results

### Study selection

**Figure 1:**
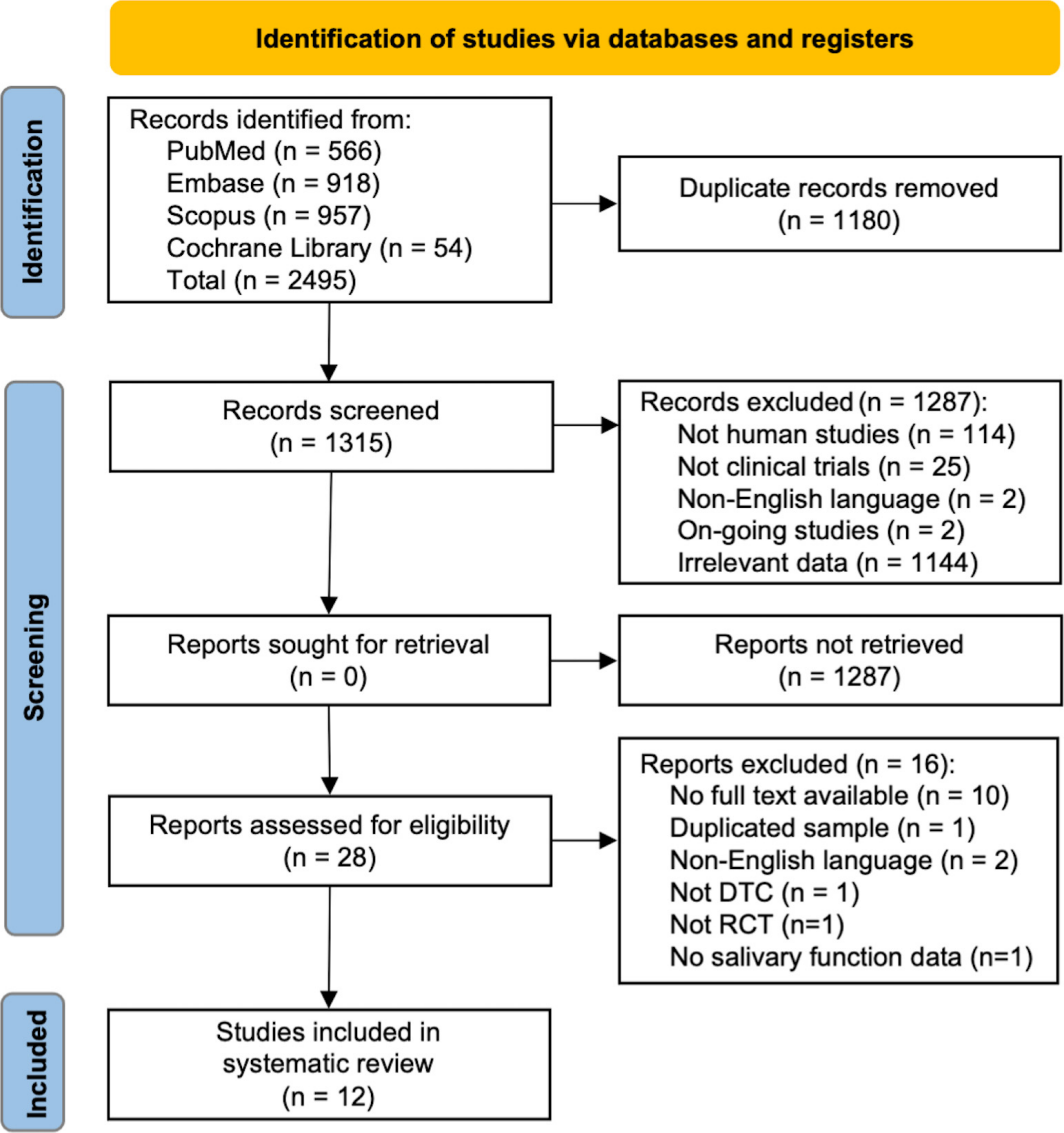
The PRISMA flow diagram.

The comprehensive literature search identified 2495 studies (**Figure 1**). After removing the duplicates, a total of 1315 studies were reviewed. Of these, we excluded 1287 studies after abstract reviewing and 16 studies after a full-text screening. Finally, twelve studies were eligible and included for analysis (**Table 1**). Due to the extensive heterogeneity in outcome measures and reporting, it was not possible to perform a meta-analysis. Therefore, a systematic review was conducted.

**Table 1:** Study characteristics.

Authors, year of publication, country	Participants (n, mean age)	I-131 dose range (mean), GBq	Interventions	Assessment methods	Results
**Parotid gland massage**					
Hong, 2014, Republic of Korea	44 patients 49.1 ± 11 y	3.7–5.6 (4.96 ± 1.07)	1) 1-min parotid gland massage2) 2-min parotid gland massage	SGS with I-123 before and after massage1) Mean change in I-123 uptake before parotid gland massage (ΔU_con_)2) Mean change in I-123 uptake after parotid gland massage (ΔU_mas_)	- ΔU_mas_ was significantly lower than ΔU_con_ in both groups (*p* < 0.001)- No significant difference of ΔU_mas_ between groups - 1-min or 2-min PG massage reduced the RAI uptake in the parotid gland and reduced parotid gland dysfunction
Son, 2019, Republic of Korea	100 patients 51 y	2.96–7.4 (NA)	1) 1-min parotid gland massage for 7 d2) Non-massageAll: sialogogue every 30 min for 4 h, 2 L of water consumption a day for 7 d	SGS with ^99m^Tc-pertechnetate at 0, 8 mo (decrease in EF >10% was defined to be abnormal)- Serum amylase at 0, 24 h- Questionnaire survey at 0, 1 d, 2 wk, 1,2,5,8 mo	- Serum amylase levels and parotid gland abnormality on SGS were significant lower in parotid gland group (*p =* 0.0052, *p* = 0.0195) - Parotid gland massage significantly reduced the incidence of salivary gland dysfunction
**Aromatherapy**					
Nakayama, 2016, Japan	71 patients 60 y	3.7–5.55 (5.31)	1) Aromatherapy group (inhale blended 1.0 mL of lemon and 0.5 mL of ginger essential oils) 2) Control (inhaled distilled water) 10 min before each meal for 2 wks during admission	SGS with ^99m^Tc-pertechnetate at d 0, 41) Change of the maximum accumulation ratio2) Change of the washout ratio	- Significant higher rate of change of accumulation ratio (*p* < 0.05) and uptake ratio (*p* < 0.05) in aromatherapy group - Aromatherapy decreased *salivary gland* dysfunction following RAI therapy
**Vitamin C**					
Liu, 2010, China	72 patients 42 y	3.7 (3.7)	Sucking vitamin C (100 mg every 4 h for 6 d) after I-131 ingestion (4 groups)1) Start at 1 h2) Start at 5 h3) Start at 13 h4) Start at 25 hAll: received 10 mg prednisolone every 8 h, at least 3 L/d of nondairy fluid, and require to stop other sialogogues	SGS images for calculate salivary absorbed dose based on the MIRD schema at 1, 2, 3, 4, 5, 13, 25, 48 h	- No significant difference of absorbed dose to the parotid glands and to the submandibular glands among the four groups - Salivary stimulation with vitamin C at any time after I-131 administration had limited effects on salivary gland function
**Vitamin E**					
Fallahi, 2013, Iran	36 patients 31 y	3.7 – 5.55(NA)	1) Vitamin E 800 IU/day at 1 wk before to 4 wk after RAI therapy 2) Control group (placebo)	SGS with ^99m^Tc-pertechnetate at 0, 6 mo1) First-minute uptake ratio (FUR) 2) Maximum uptake ratio (MUR) 3) Maximum secretion percentage (MSP) 4) Excretion fraction (EF)	The control group had;1) Significant decrease of MSP (*p* = 0.039) and EF (*p* = 0.015) of the right submandibular gland2) Significant decrease of EF (*p* = 0.035) of the left parotid gland3) Significant higher of ΔFUR and ΔEF4) Significant higher deterioration of parotid gland excretion (26.5% vs 7.9%, *p =* 0.035) - Receiving vitamin E results in less functional deterioration and was effective in protecting the salivary glands from RAI.
Upadhyaya, 2017, China	82 patients 45.6 y	3.7 (3.7)	1) Vitamin E 100 mg/d at 1 wk before to 4 wk after RAI therapy2) Vitamin E 200 mg/d at same period3) Vitamin E 300 mg/d at same period4) Control groupAll received 10 mg prednisolone every 8 h, at least 3 L/d of nondairy fluid, and require to stop other sialogogues	SGS with ^99m^Tc-pertechnetate at 0, 6 mo1) Uptake fraction (UF) 2) Uptake index (UI) 3) Excretion fraction (EF) 4) Excretion ratio (ER)	- Significant decrease in UF in control group (*p* < 0.01) - Significantly increased UI (*p* < 0.01) in group B, increased UF (*p* < 0.05) and UI (*p* < 0.01) in group C - Significant difference in ΔUF, ΔUI, ΔER between control and vitamin group (*p* < 0.05) - Vitamin E had the protective effects on salivary glands after RAI therapy.
**Selenium**					
Son, 2017, Republic of Korea	16 patients 45 y	3.7 or 5.6 (4.65)	1) Selenium 300 mcg orally for 10 d, from 3 d before to 6 d after RAI therapy 2) Control group (placebo) All: sialogogue and water consumption	Symptom questionnaire at 0, 1 wk, 6 mo Serum amylase at 0, 2d SGS with ^99m^Tc-pertechnetate at 0, 6 mo 1) Maximum uptake ratio (MUR) 2) Maximum secretion percentage (MSP) 3) Excretion ratio (ER)	- Significant lower serum amylase in selenium group (*p =* 0.009) - Significantly lower MUR, MSP, ΔMSP and EF (*p* < 0.05) in control group - Selenium is effective to reduce salivary gland dysfunction
**Amifostine**					
Bohuslavizki, 1998, Germany	50 patients 43.2 y	3 or 6 (5.5)	1) Amifostine 500 mg/m^2^ IV over 15 min before RAI therapy 2) Control group (saline) All: received vitamin C 200 mg, 1-2 L of mineral water, 40 mg dexamethasone, and 5 mg tropisetron	Xerostomia grading (WHO) SGS with ^99m^Tc-pertechnetate at 0, 3 mo 1) Uptake	- Significantly reduced uptake function in control group (*p* < 0.01) but not significantly altered in amifostine group - Higher rate of xerostomia grade I and II in control group (36% vs 0% and 8% vs 0%)- Amifostine effectively reduced salivary gland dysfunction after RAI.
Kim, 2008, Republic of Korea	80 patients 43.2 y	3.75 – 7.5 (5.5)	1) Amifostine 300 mg/m^2^ intravenously over 15 min before RAI therapy 2) Control group All: received sialogogues, additional 2 L/d of water consumption, and ondansetron 8 mg every 8 h	Xerostomia questionnaire and SGS with ^99m^Tc-pertechnetate at 0, 3, 6 mo 1) Maximal accumulation (MA) 2) Maximum secretion (MS) 3) Uptake ratio 4) T_max_	- No significant difference of functional parameters between two groups (*p* = 0.2461)- Amifostine pretreatment did not prevent the parenchymal damage to major salivary gland function after RAI.
**Pilocarpine and bethanechol**					
Silberstein, 2008, US	60 patients 47 y	NA (4.64)	1) Pilocarpine HCl 5 mg orally every 8 h for 7 d 2) Control group All: received “Cincinnati regimen” - Dexamethasone 8 mg every 12 h x 3 d - Dolasetron 1 mg every 12h x 3 d - Sialogogues while awake for 1 wk - At least 2.4 L/d of nondairy fluid	Symptoms of xerostomia at 6 – 8 mo	- No significant differences in prevalence of sialadenitis, stomatitis, xerostomia, or dysgeusia between the groups - Pilocarpine did not reduce the occurrence of radiation-associated salivary complications.
Haghighatafshar, 2018, Iran	22 patients 40.5 y	NA (5.1)	1) Pilocarpine HCl 5 mg orally every 8 h for 2 d 2) Control group (placebo)	Anterior and posterior planar images at 2, 6, 12, 24, 48 h 1) Normalized parotid count	- No significant difference in the normalized parotid count- Pilocarpine has no significant radioiodine preventive effect on parotid glands during the first 48 h after RAI therapy
Campanha, 2021, Brazil	50 patients 43 y	NA(4.63)	1) Bethanechol 25 mg orally twice a day for 1 mo 2) Control group (placebo)	Symptoms related to salivary gland dysfunction at 0, 10, 30, 90 d Unstimulated whole saliva (UWS) at 0, 10, 30, 90 dQuality of life (QoL) questionnaire at 0, 10, 30, 90 d	- Significant lower xerostomia at 10 (*p* = 0.047) and at 30 d (*p* = 0.003), and lower sialadenitis at 10 d (*p* = 0.047) in bethanechol group - No significant difference in UWS, QoL and adverse effects compared between the groups- Bethanechol reduce the incidence of xerostomia and sialadenitis in the first days after RAI.

d, day; DTC, differentiated thyroid cancer; EF, excretion fraction; h, hour; L, liter; min, minute; MIRD, Medical Internal Radiation Dose; mo, month; NA, not available; PG, parotid gland; RAI, radioactive iodine; SGS, salivary gland scintigraphy; TTD, total thyroidectomy; y, year.

### Study characteristics

The publication years of the studies ranged from 1998 to 2021. A total of 667 participants (sample size ranging from 22 to 100 participants) were included in the studies. Mean age ranged between 31 and 60 years, and I-131 dose ranged from 2.96 to 7.89 GBq. All studies were single-center studies. Nine studies were conducted in Asia (18–26), while the rest were conducted in Europe and America (27–29). Most of the studies included two experimental arms (18–20, 22, 24–29) while two studies consisted of four experimental arms (21, 23). Seven studies investigated non-pharmacological interventions (18–24), and five studies analyzed pharmacological interventions (25–29). Ten studies assessed salivary gland dysfunction by SGS (18–27), two studies evaluated xerostomia and sialadenitis symptoms (28, 29), and one study assessed quality of life (29). One study measured serum amylase (19).

### Risk of bias assessment

Using the RoB2, we found that 5 studies had a low risk of bias (20, 22, 26, 27, 29) whereas the other 7 studies had some concerns (18, 19, 21, 23–25, 28). Summary of risk of bias assessment of the 12 included trials is shown in the **Supplementary Data, Appendix 2**.

### Parotid gland massage

The external massage on the parotid gland physically squeezed or milked out the accumulated I-131 to the oral cavity. There were 2 studies (144 participants) exploring parotid gland massage in this review (18, 19). Hong et al. (18) enrolled 44 patients and randomized into parotid massage and control groups. Three serial salivary gland scans with I-123 were performed to assess the effects of the interventions (two scans performed before, and one after the intervention). The first group received two scans at a 1-minute interval (serving as control) before receiving a 1-minute parotid gland massage. The second group received two scans at a 2-minute interval (serving as control) before receiving a 2-minute parotid gland massage. The third scan was obtained immediately after the massage in both groups. The results showed that the mean value of changes in I-123 uptake after parotid gland massage (ΔU_mas_) was significantly lower than changes in I-123 uptake between control scan (ΔU_con_) for patients in both groups (*p* < 0.001), but there was no significant difference between ΔU_mas_ (*p* = 0.573) in the two groups. Thus, the researchers concluded that parotid gland massage for 1 or 2 minutes, reduced the RAI uptake in the parotid gland and potentially reduced parotid gland dysfunction. Son et al. (19) prospectively investigated the serum amylase level and SGS after I-131 therapy. A total of 100 patients were randomized into two groups (parotid gland massage and control groups). Each group simultaneously received sialogogues (e.g., lemon juice, candy, and chewing gum) ingestion every 30 minutes, starting at 4 hours after RAI therapy, and at least 2 liters of water consumption a day, while the parotid gland massage group received an additional parotid gland massage for 1 minute immediately after sialogogue ingestion. The protocol was performed at waking time for 7 days after I-131 administration. At the 8-month follow-up, serum amylase level and the ejection fraction (EF) of the salivary gland, defined as the percentage of washout relative to pre-sialogogue stimulation were assessed. A significant decrease in EF > 10% was defined to be abnormal. Serum amylase level was lower in the parotid gland massage group *(p =* 0.0052). Massage also reduced the rates of parotid gland abnormality on SGS (odds ratio, 0.3704; *p* = 0.0195).

### Aromatherapy

Only one study examined the effectiveness of aromatherapy (20). Nakayama et al. (20) included 71 patients and randomized into an aromatherapy group (inhalation of blended 1.0 mL of lemon and 0.5 mL of ginger essential oils) and a control group (inhaled distilled water). The interventions were given for 10 minutes before meal for 2 weeks during admission. The measurement of salivary gland function, change in maximum accumulation ratio, and change of washout ratio, were calculated using the time-activity curve from SGS for each salivary gland at baseline and four days after RAI therapy. The aromatherapy group showed a significantly higher rate of change in the maximum accumulation ratio (*p* < 0.05) and a significantly higher rate of change in the washout ratio (*p* < 0.05), suggesting an increase in saliva secretion after the inhalation of an essential oil containing lemon and ginger. The authors concluded that aromatherapy ameliorated salivary gland dysfunction following RAI therapy.

### Vitamin C

One study evaluated the radio-protective effects of vitamin C (21). Liu et al. (21) recruited 72 participants and randomly divided into four groups. After receiving 3.7 GBq of I-131, patients in groups A, B, C, and D began sucking vitamin C (100 mg every 4 hours in the daytime over 6 days) at 1, 5, 13, and 25 hours, respectively. All patients received 10 mg of prednisone every 8 hours, ingested at least 3 liters of nondairy liquid daily, and were required to stop taking other sialogogues except for the study vitamin C. Salivary gland absorbed dose of I-131 was assessed for I-131 washout effect of vitamin C, if the amount of I-131 secreted through saliva flow is greater than arriving blood flow, the salivary absorbed dose would be reduced. There was no significant difference in absorbed dose to the parotid glands (*p* = 0.37) and to the submandibular glands (*p* = 0.28) among the four groups. The results suggested that the intervention with vitamin C had only limited protective effects on salivary gland function.

### Vitamin E

Two studies (118 participants) explored the effects of vitamin E (22, 23). Fallahi et al. (22) enrolled 36 patients and divided into two groups. The intervention group received oral vitamin E 800 IU/day for one week before and four weeks after RAI therapy, and the control group received a placebo for the same duration. Salivary gland function was assessed using SGS at baseline and 6 months after RAI therapy. First-minute uptake ratio (FUR), the maximum uptake ratio (MUR), the maximum secretion percentage (MSP), and the excretion fraction (EF) were calculated. There were significant decreases in MSP (*p* = 0.039) and EF (*p* = 0.015) of the right submandibular gland and EF (*p* = 0.035) of the left parotid gland in the control group, suggesting decreased salivary gland functions, whereas there was no significant difference in vitamin E group. There was a significantly lower rate of change of FUR (ΔFUR) for the right parotid and a lower rate of EF change (ΔEF) for the left parotid gland in the control group compared to the vitamin E group, suggesting less functional deterioration of the parotid gland in the patients receiving vitamin E. Significant deterioration of parotid gland excretion (reduction in EF > 15%) was lower in vitamin E group compared with the control (7.9% vs. 26.5%, *p =* 0.035). The results suggested that vitamin E may protect the salivary glands from RAI among patients with DTC.

Upadhyaya et al. (23) enrolled 82 patients and randomized into four groups. Group A, B, and C received oral vitamin E 100, 200, and 300 mg/day, respectively, starting from one week before until four weeks after RAI therapy (a total of five weeks). The control group received a placebo for the same duration. Salivary gland function was assessed using SGS at baseline and 6 months after I-131 therapy. Uptake fraction (UF), uptake index (UI), EF, and excretion ratio (ER) of each salivary gland were measured. There was a significant decrease in UF in the control group (*p* < 0.01). There were significantly increased EF (*p* < 0.01) and UI (*p* < 0.05) in group A, significantly increased UI (*p* < 0.01) in group B, and significantly increased UF (*p* < 0.05) and UI (*p* < 0.01) in group C. There was a significant difference in ΔUI of the right parotid gland (*p* < 0.05), both submandibular glands (all *p* < 0.01), ΔER of the left parotid gland (*p* < 0.05), and ΔUF of the left submandibular gland (*p* < 0.05) with the trend of increasing functions in groups with higher doses of vitamin E. The results supported that vitamin E had protective effects on salivary glands after I-131 therapy.

### Selenium

One study explored the effects of selenium (24). Son et al. (24) enrolled 16 patients and divided into two groups. The selenium group received 300 mcg of selenium orally for ten days (from three days before until six days after RAI therapy). The control group received a placebo for the same period. All participants were instructed to take fluid and sialagogues such as lemon candy or chew gum after RAI therapy. The salivary gland functions were assessed by symptom questionnaires and changes of SGS parameters (e.g., maximum uptake ratio, ΔMUR; maximum secretion percentage, ΔMSP; and ejection fraction, ΔEF) at baseline and 6 months after RAI therapy. Serum amylase was measured at baseline, 2 days, and 6 months after RAI therapy. The selenium group showed significantly lower serum amylase levels (*p =* 0.009) while the control group showed significantly lower MUR, MSP, ΔMSP, and EF (*p* < 0.05), suggesting a better function of the salivary gland in the selenium group. The results suggested that selenium supplementation during RAI therapy may be effective to ameliorate salivary gland dysfunction.

### Amifostine

Two studies (130 participants) explored the effects of amifostine (25, 27). Bohuslavizki et al. (27), included 50 patients and divided them into amifostine group and control. The intervention group received intravenously amifostine 500 mg/m^2^ while the control received physiologic saline solution over fifteen minutes before RAI therapy. During admission, all patients received salivary stimulation with ascorbic acid, 40 mg of oral dexamethasone, 5 mg of tropisetron, and an addition of 1-2 liters of mineral water. Salivary gland uptake of technetium-99m pertechnetate and xerostomia were assessed at baseline and three months after RAI therapy. The uptake function was significantly reduced by 40.2% in the placebo (*p* < 0.01) but not significantly altered in the amifostine group (*p* = 0.691). The control group developed 36% and 8% of xerostomia grade I and grade II (according to World Health Organization grading system), respectively while xerostomia was not found in the amifostine group. For adverse effects, a significant decrease in mean blood pressure was observed in the amifostine group (*p* < 0.05) following the infusion, and two patients experienced circulatory collapse during the infusion. Both patients recovered without sequelae. The study suggested that salivary uptake and parenchymal function of the salivary gland can be preserved by amifostine in patients received high-dose RAI therapy.

Kim et al. (25) included 80 patients and randomly divided into the amifostine group (300 mg/m^2^ infusion before RAI therapy), and the control. All participants received 8 mg ondansetron every 8 hours and an addition of 2 liters of water during admission. SGS was performed at baseline, 3 months, and 6 months after RAI therapy. Both groups had significantly declined in functional parameters (maximal accumulation, maximal secretion, uptake ratio, and T_max_). Contrary to Bohuslavizki’s statement (27), Kim et al. (25) stated that prophylaxis with amifostine did not prevent the salivary gland dysfunction after I-131 treatment.

### Pilocarpine

Two studies (82 participants) explored the effects of pilocarpine (26, 28). Silberstein et al. (28) included 60 patients and randomly divided into the pilocarpine group (pilocarpine 5 mg orally every 8 hours for one week after RAI therapy), and the control group. All patients received 8 mg of dexamethasone and 100 mg of dolasetron 2 hours before RAI therapy and every 12 h for another 5 doses after I-131 administration. The participants were instructed to always take sugar-free hard candy or gum, gently brush the oral mucosa, and drink non-dairy fluid ≥2.4 liters/day for one week. Symptoms and signs of salivary gland dysfunction were followed within the first 10 days and 6–8 months after RAI therapy. No significant differences in the rates of sialadenitis, stomatitis, subjective xerostomia, or taste disorder was found between the pilocarpine and placebo at the 6-month follow-up.

Haghighatafshar et al. (26) enrolled 22 patients and divided into pilocarpine and control groups. The patients received oral pilocarpine 5 mg or placebo every 8 hours for 48 hours after RAI therapy. The efficacy was assessed by parotid uptake at 2, 6, 12, 24, and 48 hours after RAI administration. No significant difference was found in the normalized parotid count between the groups. This study concluded that pilocarpine had no significant radioiodine preventive effect on parotid glands during the first 48 hours after I-131 administration.

### Bethanechol

Only one study explored the effects of bethanechol (29). Campanha et al. (29) included 50 patients and randomized into two groups. Patients received bethanechol (2 mg orally twice a day) or placebo for one month after RAI therapy. Both groups were compared at baseline, 10, 30, and 90 days of symptoms related to salivary gland dysfunction, unstimulated whole saliva (UWS), and quality of life questionnaire. The bethanechol group reported significant lower xerostomia at 10 days (*p* = 0.047) and 30 days (*p* = 0.003), and significant lower sialadenitis symptoms at 10 days (*p* = 0.047). No difference in UWS, quality of life, and adverse effects was demonstrated between the groups. Bethanechol therapy was generally well tolerated. Bethanechol may reduce the incidence of xerostomia and sialadenitis on the first day after RAI therapy.

## Discussion

We systematically reviewed the effectiveness of non-pharmacological and pharmacological interventions in the prevention of salivary gland dysfunction following RAI therapy for DTC. The analyses showed that parotid gland massage, aromatherapy, vitamin E, selenium, and bethanechol showed a significant reduction in salivary gland dysfunction induced by RAI therapy, while the benefits from amifostine had inconsistency among studies. On the contrary, vitamin C and pilocarpine showed no significant protective effects on salivary gland dysfunction. **Table 2** provides an overall summary of the review.

**Table 2:** Summary of outcomes.

Intervention Type	Number ofrandomized controlled studies	Number of Outcomes	
		Positive	Negative
Parotid gland massage	2	2	0
Vitamin E	2	2	0
Aromatherapy	1	1	0
Bethanechol	1	1	0
Selenium	1	1	0
Amifostine	2	1	1
Vitamin C	1	0	1
Pilocarpine	2	0	2

Nonpharmacological therapy including parotid gland massage and aromatherapy can prevent the damage caused to the salivary gland from RAI. The external massage on the parotid gland can be useful to prevent the salivary gland injury by reducing radiation exposure from physically milking out the accumulated radioisotope in the salivary gland and duct to the oral cavity (30). Two RCTs showed that parotid gland massage for 1-2 minutes had a significant preventive effect on RAI therapy (18, 19). Aromatherapy has the potential ability to increase saliva production during inhalation and appeared to demonstrate efficacy in preventing RAI-related salivary gland dysfunction (20). This result was only reported by one study, so it should be replicated in future studies. *via* Salivary flow can be stimulated using sialogogues. The use of sour taste stimulation (such as vitamin C) after I-131 administration can increase the salivary flow and accelerate I-131 washout from the salivary gland. Thus, previous prospective non-randomized study, such as that of Nakada et al. (31) recommended saliva stimulation by lemon candy 24 hours after I-131 administration. However, the study showed a significantly higher rate of sialadenitis in the early lemon candy sucking group (1 hour after I-131) than the late sucking group (24 hours after I-131) (64% and 37%, respectively, *p* < 0.05). Jafari et al. (32) demonstrated that vitamin C had a radio-protective effect against oxidative stress regardless of the time of administration before or after RAI therapy. In addition, the greater benefits had been demonstrated in the group that received treatment before RAI therapy, suggesting that vitamin C had a protective effect rather than attenuating effect. However, this study reported the antioxidant effects of vitamin C but did not study the direct effects on the salivary glands. Also, salivary gland function did not demonstrate a similar improvement in the other study (21). These conflicting results from the above-mentioned studies could not be directly compared because the first study measured serum oxidative stress markers (32), but another study assessed salivary function by scintigraphy (21). Therefore, the further research is needed to explore the potential protective effects of vitamin C.

The other antioxidants such as vitamin E, selenium, and amifostine showed potential benefits in reducing salivary gland dysfunction from RAI, although results are limited by a small number of included studies. In patients who underwent external beam irradiation against oral cancer, a significant improvement of the salivary flow and properties was demonstrated in the vitamin E group compared with the control (33). Two RCTs included in this review showed significant radio-protective effects of vitamin E. Fallahi et al. (22) reported that vitamin E has a radio-protective effect from RAI therapy among DTC patients. However, the study had a small sample size with a single-dose administration of vitamin E. Upadhyaya et al. (23) further investigated with larger sample sizes and multiple doses of vitamin E (100, 200, and 300 mg/day), and revealed a greater protective effect occurred in the groups with higher doses of vitamin E. However, there was no direct comparison between the vitamin E groups. Larger studies are needed to confirm these results.

Selenium is an essential trace element that plays a crucial role in the reproduction, thyroid hormone metabolism, DNA synthesis, and protection the cells against oxidative stress (34, 35). Selenium showed limited effects in the prevention of loss of taste from external radiation of head and neck cancer (36). In contrast, the selenium group had reduced the radiation damage, evidenced by lower serum amylase levels and higher salivary function parameters than the control group in a study with DTC patients treated with RAI therapy (24). However, the number of participants (8 participants/arm) was too small to conclude the results.

Amifostine is an organic thiophosphate prodrug which is dephosphorylated to its active metabolite WR-1065 which acts as a potent intracellular scavenger of oxygen free radicals. It has been shown to selectively protect normal tissues (without protecting tumor tissue) from the toxicity associated with chemo-radiotherapy (37). Amifostine is the only one radio-protective medication that has been approved by the U.S. Food and Drug Administration. Given intravenously amifostine with dexamethasone and vitamin C was more effective than placebo in reducing salivary gland dysfunction in patients who received high-dose RAI (27). However, no radio-protective effect on salivary glands was demonstrated in the larger sample-sized study (25). The systematic review by Ma et al. (38) analyzed the protective effects of amifostine on RAI-associated sialadenitis by using data from two RCTs (25, 27) and suggested that amifostine has no significant protective effects.

Pilocarpine is a parasympathomimetic agent that can stimulate muscarinic receptors in salivary gland and increases the salivary secretion. Pilocarpine has been approved for the treatment of xerostomia following radiation therapy for the head and neck cancer (39). As a treatment, a previous prospective nonrandomized study (not included in this systematic review) revealed that pilocarpine has beneficial effects in patients with salivary gland impairment after RAI (40). But the two RCT studies included in this review did not demonstrate a benefit on the RAI preventive effect on parotid glands (26, 28). The negative results may be explained by the strong sialorrheic effect of the Cincinnati regimen in both intervention groups (28). The intense sialorrheic management called Cincinnati regimen consists of oral sialogogues, oral hydration, a serotonin receptor blocker, and dexamethasone. In addition, another RCT study showed pilocarpine had no significant RAI protective effect on the parotid glands (26). These studies had a small number of patients. Further research is needed to provide evidence of the benefits of pilocarpine in reducing salivary gland disorders from RAI therapy. The other cholinergic agonist, bethanechol is the carbamic acid ester of 2-methylcholine which can stimulate the muscarinic receptor. Bethanechol has its applicability to decreasing the salivary gland dysfunction in head and neck cancer patients receiving radiation therapy (41, 42). A recent RCT study showed that prophylactic therapy of bethanechol can significantly lower incidences of acute sialadenitis and xerostomia in patients who underwent RAI therapy (29). The different results between pilocarpine and bethanechol (29) may be explained by the different duration of intervention. Pilocarpine was administered for 48 hours-1 week after RAI therapy (26, 28) while receiving a longer duration of bethanechol for 1 month after RAI therapy (29).

There are several limitations to this systematic review. First, most studies have a small number of participants ranging from 22 to 100. Second, the studies did not refer to the blinding of subjects, which increased the chance of open-label bias. Third, in some studies, the participants were instructed to take fluid and sialagogues after RAI therapy promoting I-131 secretion. These may be unintended benefits and masked any small additional benefits of the intervention. Fourth, there was heterogeneity in the intervention methods and the assessments of salivary gland function. Therefore, a meta-analysis was not performed. Fifth, the risk factors associated with salivary gland dysfunction, such as the dose of RAI (7) and the degree of disease involvement in the neck (43) might have not be well-balanced among the RCTs. Finally, we did not include other types of clinical trials such as retrospective studies, cohort studies, non-RCTs, and studies published in languages other than English. Most studies were conducted in Asia (18–26). However, no evidence suggested that the frequency of sialadenitis is related to ethnicity or geographic area.

## Conclusions

This systematic review analyzed the best available evidence from previous RCTs and suggested that parotid gland massage, aromatherapy, vitamin E, selenium, amifostine, and bethanechol may have benefits on reducing salivary gland dysfunction from RAI therapy in DTC patients, while vitamin C and pilocarpine may not be beneficial. The number of studies included in this systematic review was relatively small. Further well-designed large-scale RCTs are needed to precisely evaluate the interventions in the prevention of salivary gland dysfunction following RAI therapy for DTC.

## Data availability statement

The original contributions presented in the study are included in the article/**Supplementary Material**. Further inquiries can be directed to the corresponding author.

## Author contributions

AA-A, SR, and CS contributed to conception and design of the study. AA-A, TA, SR, and CS designed the methods. AA-A, JS, and CS reviewed literature, collected data, contributed to data analysis. AA-A wrote the first draft of the manuscript. TA, SR, and CS advised on the review and reviewed the final manuscript. All authors contributed to manuscript revision and approved the submitted version.

## Conflict of interest

The authors declare that the research was conducted in the absence of any commercial or financial relationships that could be construed as a potential conflict of interest.

## Publisher’s note

All claims expressed in this article are solely those of the authors and do not necessarily represent those of their affiliated organizations, or those of the publisher, the editors and the reviewers. Any product that may be evaluated in this article, or claim that may be made by its manufacturer, is not guaranteed or endorsed by the publisher.

## Abbreviations

DTC, differentiated thyroid cancer; RCT, randomized controlled trial.
