# Supplementary material for: Prevention of salivary gland dysfunction in patients treated with radioiodine for differentiated thyroid cancer: A systematic review of randomized controlled trials
Source: Front Endocrinol (Lausanne). 2022 Aug 29;13:960265. doi: 10.3389/fendo.2022.960265 (PMC9465079; doi:10.3389/fendo.2022.960265)
Supplement: Supplementary file 1 [file Table_1.docx]

Supplementary Material

**Appendix 1:** Search terms (November 11, 2021)

**PubMed:** 566 citations

(radioiodine OR radioactive iodine OR iodine-induced OR ¹³¹I) AND (sialadenitis OR sialoadenitis OR salivary gland OR aromatherapy OR lemon candy OR vitamin E OR vitamin C OR massage OR selenium OR amifostine OR pilocarpine OR chewing gum)

**Scopus:** 957 citations

TITLE-ABS-KEY ( ( radioiodine  OR  radioactive  AND  iodine  OR  iodine-induced  OR  ¹³¹i )  AND  ( sialadenitis  OR  sialoadenitis  OR  salivary  OR  aromatherapy  OR  "lemon candy"  OR  "vitamin E"  OR  "vitamin C"  OR  massage  OR  selenium  OR  amifostine  OR  pilocarpine  OR  chewing-gum ) )

**EMBASE:** 918 citations

(radioiodine OR 'radioactive iodine' OR 'iodine induced' OR ¹³¹i) AND (sialadenitis OR sialoadenitis OR 'salivary gland' OR aromatherapy OR 'lemon candy' OR 'vitamin e' OR 'vitamin c' OR massage OR selenium OR amifostine OR pilocarpine OR 'chewing gum')

**The Cochrane Library:** 54 citations

(radioiodine OR radioactive iodine OR iodine-induced) AND (sialadenitis OR sialoadenitis OR salivary gland OR aromatherapy OR lemon candy OR vitamin E OR vitamin C OR massage OR selenium OR amifostine OR pilocarpine OR chewing-gum) in All Text - (Word variations have been searched)

**Appendix 2:** Quality assessment of the included studies using the RoB2, a revised Cochrane risk-of-bias tool for randomized trials. RoB2 is structured into 5 domains through which bias might be introduced into the result. These were identified based on both empirical evidence and theorical considerations. The five domains for individually randomized trials are: (1) bias arising from the randomization process; (2) bias due to deviations from intended interventions; (3) bias due to missing outcome data; (4) bias in measurement of the outcome; and (5) bias in selection of the reported result.

| Study | Randomization | Deviation from the intended interventions | Missing outcome data | Measurement  of outcome | Selection of the reported result | Overall |
| --- | --- | --- | --- | --- | --- | --- |
| Bohuslavizki, 1998 | LR | LR | LR | LR | LR | LR |
| Campanha, 2021 | LR | LR | LR | LR | LR | LR |
| Fallahi, 2013 | LR | LR | LR | LR | LR | LR |
| Haghighatafshar, 2018 | LR | LR | LR | LR | LR | LR |
| Hong, 2014 | LR | SC | LR | LR | LR | SC |
| Kim, 2008 | LR | SC | LR | LR | LR | SC |
| Liu, 2010 | LR | SC | LR | LR | LR | SC |
| Nakayama, 2016 | LR | LR | LR | LR | LR | LR |
| Silberstein, 2008 | LR | SC | LR | LR | LR | SC |
| Son, 2017 | LR | SC | LR | LR | LR | SC |
| Son, 2019 | LR | SC | LR | LR | LR | SC |
| Upadhyaya, 2017 | LR | SC | LR | LR | LR | SC |

LR: low risk of bias; SC: some concerns; HR: high risk of bias
